# Looking for the Brain Inside the Initial Teacher Training and Outreach Books in Portugal

**DOI:** 10.3389/fpsyg.2022.737136

**Published:** 2022-02-28

**Authors:** Joana R. Rato, Jorge Amorim, Alexandre Castro-Caldas

**Affiliations:** Centre for Interdisciplinary Research in Health, Institute of Health Sciences, Universidade Católica Portuguesa, Lisbon, Portugal

**Keywords:** syllabus, teacher training courses, neuroscience education, outreach books, mind, brain, and education, rapid review

## Abstract

The fascination with brain research is widespread, and school teachers are no exception. This growing interest, usually noticed by the increased supply of short-term training or books on how to turn the brain more efficient, leads us to think about their basic training and outreach resources available. Little is known about what the official Initial Teacher Training (ITT) offers concerning the brain literature and if it meets scientific standards. Also, what are the science communication materials that teachers can access to learn about the developing brain remain undiscussed. First, we examined the ITT courses taught in Portuguese Higher Education, both in public and private institutions, to identify the syllabus with updated neuroscientific knowledge. Second, we searched for the neuroscience-related books published in the last 6 years through the National Library of Portugal database. Thirty ITT courses and 35 outreach publications were reviewed through a rapid review methodology. Our results showed an absence of curricular units indicating in their programs that brain research, and its relationship with learning, would be taught in a representative and updated way. In contrast, the number of brain-related books for educators increased in Portugal, corroborating the demand for this field of study by these professionals. Based on the literature that shows how misunderstandings about the brain have increased in school contexts, our discussion recognizes that science outreach could be a way to increase the scientific literacy of school teachers with the research community working more in this direction, but, since a previous problem seems to be unsolved, there is an urgent need for specialized attention to the development of training curricula for future kindergarten and elementary school teachers.

## Introduction

In the educational neuroscience field, which has been advancing more to emerge as a distinct discipline, how to integrate neuroscience into educational practice remains a discussion (Wilcox et al., 2021). Between the past (or still present) caution views and promising research works arising, all agree that more is needed to build a robust translational bridge between brain research and classroom practices (e.g., Bruer, 1997, 2006; Ansari and Coch, 2006; Kelleher and Whitman, 2018; Tan and Amiel, 2019).

One of the problems pointed on this highly interdisciplinary research framework is based on the scarce specialty literature. It has been presenting more discussion about the promise of applying neuroscience to education than educational neuroscience applications already studied (Bruer, 2016). Increasing the evidence-based practice (EBP) rate in school contexts is a pre-occupation in the educational sciences since the 1990s (Davies, 1999). Despite the growing of EBP potential recognition to transform teaching and learning, incorporating collaborative research projects into everyday school practice has not been seen by the teachers as so easy as expected (Walker et al., 2019).

The relationship between neuroscience and education advances theoretically but not practically in natural contexts (Thomas et al., 2019), and we still found an ongoing debate about the potential of interdisciplinary research and its applications. Several societies and special interest groups established being the International Mind, Brain and Education Society one of the first to be released and counting currently with 17 years of existence (IMBES^1^).

The enthusiasm of educators and policymakers to support their educational policies and practices with scientific evidence quickly caught the attention of the commercial companies to sell new learning techniques with science make-up (Goswami, 2006; Howard-Jones, 2014b). Teachers need to know how to disentangle whether what arrives at school comes from good scientific sources or pseudoscience, for less attentive or empowered teachers easily can be dragged along by speculative ideas, interfering with their pedagogical decision-making. Even with some notions of how we process information, it is not enough to understand how brain mechanisms work, and it may lead to erroneous theories about brain functioning (Thomas et al., 2019). Some assumptions can also be out of initial training and be more connected to popular contexts and everyday interpretations (Schregel and Broer, 2020). We knew that short-term training can impact personal beliefs and promote awareness about myths but do not develop full immunity to neuromyths (McMahon et al., 2019). People can have a profound interest in a topic and dive into non-scientific sources about it, and some popular courses about the brain can reinforce neuromyths (Hughes et al., 2020).

According to previous surveys, despite interest of teachers in brain topics, a high percentage believed in neuromyths. Moreover, this happens despite they are teachers from Portugal, Spain, England, The Netherlands, Turkey, or China (Dekker et al., 2012; Rato et al., 2013; Howard-Jones, 2014a; Ferrero et al., 2016). These studies illustrate how teachers will not always be explicitly aware of whether it is or not an evidence-based source. A recent systematic review focusing on the neuromyths popularity in educational contexts showed that brain misunderstanding is remarkably consistent among worldwide teachers and highlighted the need to improve the scientific content in higher education and the importance of in-service teacher training (Torrijos-Muelas et al., 2021).

What contributes the most to these beliefs is not yet consensual, and data suggest that factors like age, education level, and neuroscience exposure influence neuromyths detection (Macdonald et al., 2017). Hence, it is not surprising that greater knowledge exchange in the context of teacher training already has been promoted by diverse international bodies (Coch, 2018). Examples of this are the Royal Society in the United Kingdom (2011), the International Society for Neuroscience (2009), and the Organization for Economic Co-operation and Development (2007), which all of them presented recommendations that preparation programs must include neuroscience components relevant to educational issues since we already have brain knowledge that should be central to the teacher and could provide to him another perspective on learning, development, and instruction (Leibbrand and Watson, 2010).

Knowing that for any professional domain, initial training plays a fundamental role in the success of the practice; we developed this study to verify which updated scientific knowledge coming from the neurosciences field can be accessed by teachers in their basic training. First, we analyzed the syllabus in the Portuguese reference courses for teacher education. And second, we surveyed the outreach books about the brain available in Portuguese and published in Portugal in recent years.

## Materials and Methods

Based on a double objective, we divided the study into the (1) Initial Teacher Training (ITT) syllabus survey and the (2) neuroscience outreach books rapid review.

### Mind-Brain Curricular Units Present in the Teacher Training Courses

The Portuguese Elementary Education Degree (in Portuguese, *Licenciatura em Educação Básica*) is a 3-year course directed to prepare future professionals to deal with children from 0 to 12-year-old in school contexts. This ITT course is required to access the master’s degree that enables later to teach pre-school and elementary years (Faria et al., 2016). It is the basic training for any future teacher and includes a total of 180 European Credit Transfer and Accumulation System (ECTS) credits. Through the DGES Database (Direção Geral do Ensino Superior^2^), we found the list of the higher school institutions and through their web pages (Supplementary Appendix 1); we scanned the public information. Thirty (*N* = 30) elementary education ongoing courses in the academic year 2020/2021 were reviewed to identify the curricular units related to the mind-brain scientific research domain. We used the following criteria for the analysis of the courses: name of the curricular unit, contents covered, unit objectives, and recommended bibliography. Unrelated curricular units, i.e., without mind-brain topics, were excluded from our selection process. Since the analysis was only based on publicly available information, the data limitations in several courses prevented us from relying on bibliographic criteria for robust conclusions. The main reason for selecting the courses for Early Childhood Education and Elementary Education is because these teachers will be the first line of contact between children and formal education, in which the importance of the early years in human development must be specially attended to in your training.

### Books About the Brain Published in Portuguese

To review the books available with a brain subject focus, we used the National Library of Portugal online database (BPN---Biblioteca Nacional de Portugal^3^). The mission of the BPN is to gather, protect, and make available all knowledge in the Portuguese territory. With 200 years of existence, BNP acts as the National Bibliographic Agency and gathers its collection either through legal deposit or through the acquisition of works of a recognized bibliographic or cultural value, keeping a collection that exceeds 3 million publications (Biblioteca Nacional de Portugal, 2021). We defined a procedure similar to the systematic review for eligibility criteria, but as we were limited to a single database (i.e., BNP database is the only one with the national collection of this type of publications), we used a rapid review format for a quantitative approach (Grant and Booth, 2009). In records and titles in European Portuguese from 2015 to 2020, we collected the data with the terms including, brain, neuroscience, and neuropsychology using the advanced search option that allowed the use of Boolean operators (e.g., AND; OR). The database advanced option also allowed to limit the search to a specific catalog (i.e., science, educational, and outreach items), the years of publication, and personalize data output. The selection criteria for the book search were first based on the title (i.e., descriptors combination), then the summary, and index reading. The background of the authors was taken into account (if were from academia/clinician specialist in the field or not) acting as a myth-screening process. We excluded the academic thesis, non-dissemination books related to brain-mind themes (e.g., health legislation and national health reports), and books written in languages other than Portuguese. The terms selected simulate a basic search to learn about this main topic since it will also be the word brain or neuro-prefixes, the most searched for on the covers of books. The time frame of 6 years was defined to follow the same period of the revised initial teacher training courses and coincide with the latest government changes regarding curricula in Portuguese higher education. Three researchers were involved in the selection process, one screened each record, the other screened the list for a tiebreaker, and the third reviewed the final list obtained to check eligibility decisions.

## Results

Concerning the ITT courses, we found 30 open courses in Portugal among which, 20 are from public higher education and 10 from private institutions (Table 1). To understand the representativeness of the curricular units in the mind-brain domains, we analyzed the number of ECTS and the proportion considering the total of 180 ECTS for training completion. Based on the data that stand out, we figured out that the ISEC Lisboa is the higher institution that makes more investment in these domains presenting 10% of the required ECTS. The standard curricular unit in practically all courses is Developmental Psychology.

**TABLE 1:** Initial Teacher Training (ITT) courses and European Credit Transfer and Accumulation System (ECTS) distribution by the curricular units with mind-brain subject domain (*N* = 30).

			ECTS by scientific domain						
		Higher school institutions	Developmental Psychology	Educational Psychology	Human Biology	Psycholin- guistics	Total	%/180 ECTS	Access grade (M)
Public	1	University of Algarve	0	3	0	0	3	1.7	12.77
	2	University of the Azores–Faculty of Social and Human Sciences	3	0	6	0	9	5.0	11.76
	3	University of Aveiro	0	6	0	0	6	3.3	13.30
	4	University of Évora–School of Social Sciences	5	0	0	0	5	2.8	-
	5	University of Madeira–Faculty of Social Sciences	0	3	0	0	3	1.7	11.35
	6	University of Minho	5	0	5	0	10	5.6	13.26
	7	University of Trás-os-Montes e Alto Douro	0	0	0	0	0	0	12.18
	8	Polytechnic Institute of Beja–Higher School of Education	0	5	0	0	5	2.8	12.26
	9	Polytechnic Institute of Bragança	4	0	0	0	4	2.2	10.86
	10	Polytechnic Institute of Castelo Branco	3	3	0	0	6	3.3	11.27
	11	Polytechnic Institute of Coimbra	2	2	0	0	4	2.2	13.73
	12	Polytechnic Institute of Guarda	4	0	0	0	4	2.2	9.85
	13	Polytechnic Institute of Leiria–Higher School of Education	3	0	0	0	3	1.7	10.89
	14	Polytechnic Institute of Lisboa–Lisbon Education College	5	0	0	0	5	2.8	13.68
	15	Polytechnic Institute of Portalegre	4	0	0	0	4	2.2	-
	16	Polytechnic Institute of Porto–Higher School of Education	5	0	0	0	5	2.8	14.52
	17	Polytechnic Institute of Santarém–Higher School of Education	0	4	0	0	4	2.2	12.10
	18	Polytechnic Institute of Setúbal	4	0	0	0	4	2.2	11.28
	19	Polytechnic Institute of Viana do Castelo	5	0	0	0	5	2.8	11.16
	20	Polytechnic Institute of Viseu–Higher School of Education	4	0	0	0	4	2.2	11.23
Private	1	ISPA–University Institute of Psychological, Social and Life Sciences	6	0	0	0	6	3.3	-
	2	Higher School of Education of Fafe	6	0	0	0	6	3.3	-
	3	Jean Piaget Higher School of Education of Arcozelo	3	0	0	0	3	1.7	-
	4	João de Deus Higher school of Education	5	0	5	0	10	5.6	-
	5	Paula Frassinetti Higher School of Education	3	3	0	0	6	3.3	-
	6	Jean Piaget Higher School of Education of Almada	4	0	0	0	4	2.2	-
	7	Polytechnic Institute of Lusofonia–Higher School of Education	3	0	0	7	10	5.6	-
	8	Higher Institute of Educational Sciences	3	0	0	0	3	1.7	-
	9	Higher Institute of Educational Sciences of Douro	3	0	0	6	9	5.0	-
	10	ISEC Lisboa–Higher Institute of Education and Sciences	3	3	6	6	18	10.0	-
		Total	95	32	22	19	168		

According to the list of applications for the first phase in 2020, the ITT courses only filled 71.3% (*N* = 630) of the open vacancies (*N* = 846). Despite our 20 public courses list, the official results of the entry grade average in higher education only count to 19. For reasons beyond our knowledge, the University of Évora does not appear. The grades (ranking between 0 and 20) of the last student admitted in the 19 public courses (*M* = 12.01; SD = 1.17) show the highest grade was 14.52 (Polytechnic Institute of Porto—Higher School of Education), and the lowest grade was 9.85 (Polytechnic Institute of Guarda—Higher School of Education, Communication, and Sports). Seven courses have filled all student vacancies, and four courses have less than five students each (DGES, 2020). In private institutions, this classifications entry system does not apply.

Of the 30 courses reviewed, we found 46 curricular units linked to human mind themes. No unit names were found with the prefix neuro (neither the term brain) or that explicitly addressed the link between neuroscience and cognition in the contents or objectives of the curricular unit (e.g., how the nervous system enables cognition). The selected units fall into the course general education category and were distributed across four major domains (Table 2). The available syllabus shows us that brain-based concepts are scarce, and the “mind-brain” domains only have a visible presence through classical theories about mental development (e.g., Piaget, Vygotsky). In the case of “Human Biology” and “Psycholinguistics,” we only inserted the syllabus for the count whose we could verify that were within the mind-brain themes. While the psychology curricular units were more consensual about the topics approached, “human biology” without reference to the study of the nervous system and “linguistics” without the study of brain activity and structures were excluded from our final list.

**TABLE 2:** Selected curricular units with mind-brain approach in the Initial Teacher Training (ITT) courses (*N* = 46).

Curricular Units	*Psychology of Development and Learnin g*	*Educational Psychology*	Human Biology*	Language Acquisition and Development*
	Developmental Psychology I and II	*Psychology of Education*	*Human Biology and Health*	Portuguese and Language Aquisition
	Childhood and Adolescence Psychology	Foundations of Educational Psychology	Human Biology and Health Promotion	Reading and Writing Psychogenesis*
	*Child Psychology*		Human Body and Health	Language, Cognition, and Plurilingual*

Main scientific domain	Developmental psychology	Educational psychology	Human biology	Psycholinguistics
	(*n* = 26)	(*n* = 9)	(*n* = 5)	(*n* = 6)

*Optional unit; in italics more than one record.

**FIGURE 1:**
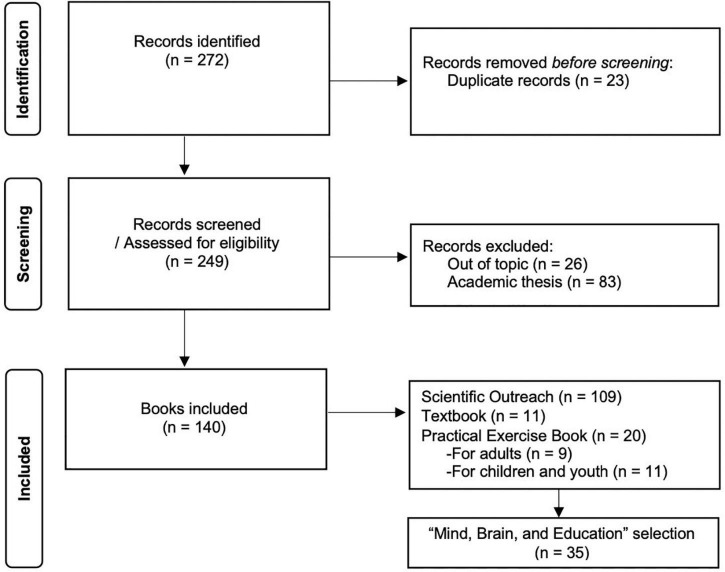
Flow diagram of the brain topic publications via the database of the National Library of Portugal.

As for the survey of books about the brain, applying our search descriptors, the BNP database showed us 272 records (Figure 1). Considering the exclusion criteria previously defined, we excluded a total of 132 records related to (i) academic thesis, (ii) duplicates (first edition only considered), (iii) non-scientific outreach books (e.g., health legislation and national health reports), and (iv) non-Portuguese written books.

We included 140 records from which we carried out a screening to classify the books to further fine the selection. As for the original language of the books, only 19% are written by Portuguese authors, in which the majority (81%) are translations from English, Spanish, and Italian. From our classification based on the BPN descriptions, we identified 78% outreach books, 8% textbooks, 6% exercises books for adults, and 8% for children/youth (i.e., brain-training exercise books). Within the outreach category, we looked into the books within the scope of the mind, brain, and education themes (*N* = 35).

**FIGURE 2:**
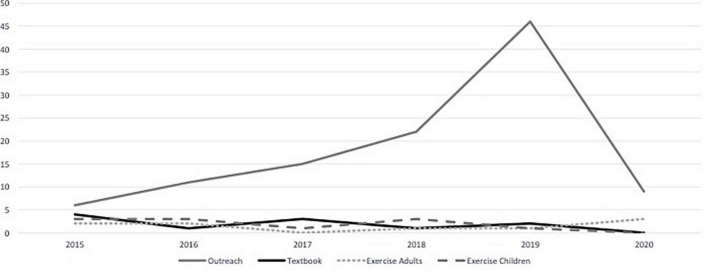
Brain-related books published (2015–2020).

The publication number shows an increasing trend over the last 6 years, with 2019 standing out compared with other years (Figure 2). In the distribution of percentages per year, 11% of publications were found in 2015, growing 1% in each of the following 2 years, reaching 19% in 2018 and 36% in 2019. In 2020, it drops to 9%, and we verified that this break in the growing trend is probably due to the mandatory stop of the publishers caused by the COVID-19 pandemic. However, the 2019 difference also could be related to the Neuroscience and Psychology collection, where only this year, 16 books were distributed by a Portuguese weekly magazine of general information. Between 2015 and 2020, we found that the books *Open brain!* (2015), *The child’s brain explained to parents* (2016), and *The brain: a story of you* (2017) were the top three books reprinted in Portugal, assuming a higher sales volume.

Within the included books list (*N* = 140), we selected those that presented valuable content for teachers to learn about the neuroscience of learning, reading, identity, language, memory, and attention (*N* = 35). Table 3 presents our mind, brain, and education selection books describing a brief content analysis of these publications. Most of the 35 selected books (Damásio, 2015; Searle, 2015; Bilbao, 2016; Castro Caldas, 2016; Cotrufo, 2016, 2019; Dierssen, 2016, 2019; Sena, 2016; Fonseca, 2017; Rato and Castro Caldas, 2017; Rego et al., 2017; Lauffer, 2018; Matute, 2018; Sigman, 2018; Alonso, 2019; Berardi, 2019; Bote, 2019; Burgaya-Márquez, 2019; Canessa, 2019; Caruana, 2019; Daphna, 2019; Domínguez, 2019; Garcia, 2019; Maojo, 2019; Quintero and Álamo, 2019; Sepulcre, 2019; Tafet, 2019; Viosca, 2019; Castro Caldas and Rato, 2020; Correia and Ferreira, 2020) are written by academics or clinicians in the fields of medicine, neuroscience, and psychology, with four exceptions to report (10, 11, 13, 15, refs number in Table 3). The guide for discovering the brain by Martins et al. (2017), was written by the writers of children books; however, the contents were reviewed by a panel of scientific experts. The book about how students can get better grades (Gaspar, 2018) is authored by a math teacher responsible for the Neurosup project to help French students and teachers learn better using neurosciences, although no scientific evidence on the impact of the project was found so far. Two books (10, 15) were written by non-scientific background writers (Gifford, 2017; Ibánez, 2018) with experience in outreach for a broader public.

**TABLE 3:** Outreach books selection with mind, brain, and education themes (*N* = 35).

Year		Author(s)	Books title in English (title in the Portuguese edition)	Content
2015	1	Searle, John R.	Mind, brain and science (Mente, cérebro e ciência)	A philosophy approach about mind, body, and consciousness as a function of the brain. Intending to connect common sense knowledge and scientific research data and reflection it also introduces the reader to the main problems of the philosophy of the mind.
	**2**	Damásio, António	Descarte’s error (O erro de Descartes: emoção, razão e cérebro humano)	A 1995 bestseller that is re-released in an updated version. It is an invitation to a journey of discovery of the connection between emotion and reason. also serves as an introduction to modern cognitive neuroscience.
2016	**3**	Castro Caldas, Alexandre	Life of the brain (A vida do cérebro: da gestação à idade avançada)	An invitation to learn about the brain from its formation in the embryo, through the first months of the baby’s life, then entering the period of adolescence, passing through adulthood, and ending at a more advanced age.
	**4**	Sena, Armando	Brain, health and society (Cérebro, Saúde e sociedade)	An introductory textbook about the brain with perception and memory descriptive chapters.
	**5**#	Dierssen, Mara	The artistic brain: creativity and neuroscience (O cérebro artístico: a criatividade segundo a neurociência)	With the resources humans used in Arts, what is the biological meaning of it? Years and years of the creation of beautiful pieces of art as a means of expression. Our species created patterns of shapes, light, colors, and symbols. This book is an introduction to the analysis of neuroscience on human art.
	**6**#	Cotrufo, Tiziana	The brain and the emotions (O cérebro e as emoções: sentir, pensar, decidir)	Outreach book about the biological functioning of emotions.
	7	Bilbao, Álvaro	The child’s brain explained to parents (O cérebro da criança explicado aos pais)	Presented as a practical manual that summarizes the knowledge that neuroscience could provide to parents and educators to help children achieve full intellectual and emotional development.
2017	8	Fonseca, Vítor	Neuropsicomotricity: essays about the relationship between body-motricity-brain-mind (Neuropsicomotricidade: ensaio sobre as relações corpo-motricidade-cérebro-mente)	The study of the connections between body, brain, mind, and motricity has improved with the research in neuroscience and neuroimaging. Research on these topics goes deep since the beginning of our species evolution. This book is a narrative perspective of our development as a species focusing on the action that shaped our mind and brain.
2017	9	Rego, Ana Cristina; Duarte, Carlos; Oliveira, Catarina	Neurosciences (Neurociências)	Neuroscience textbook is written for college students. It addresses topics, such as the central and peripheral nervous system, neurotransmission processes, the cellular, and molecular bases that determine the formation of memory, the dysfunctional and pathological processes associated with stress, and neuropsychiatric diseases, among other topics that underlie a basic neuroscience curricular unit.
	10	Gifford, Clive	The Human brain in 30 seconds (O cérebro em 30 segundos)	Each topic is presented in a neat 30-s soundbite, supported by a 3-s flash summary and full-page, colorful illustration. Active “missions” support the topics and encourage children to find out more. The attention-grabbing format is engaging and immediate, introducing readers aged from eight up to this part of their bodies called the brain.
	**11**	Martins, Isabel Minhós Pedrosa, Maria M.	Inside. Guide for discovering the brain (Cá Dentro. Guia para descobrir o cérebro)	Designed to satisfy the curiosities about the working of the mind and brain. It’s an illustrated book, written and designed with the collaboration of a team of psychologists and researchers. This is a book aimed at children and youth.
	**12**	Rato, Joana; Castro Caldas, Alexandre	When your son’s brain goes to school (Quando o cérebro do seu filho vai à Escola: boas práticas para melhorar a aprendizagem)	There’s a growing desire to apply neuroscience in education, but science moves at a different speed than expectations. In this science outreach work, the authors promote the research conducted scientifically and highlight the study of the learning brain.
2018	13	Gaspar, Éric	How do get better grades at school? (Como ter as melhores notas da escola: o cérebro e os seus truques: é fácil conseguires!)	How to memorize better to get better grades at school? This book is a fun guide to improve school grades, with strategies based on neuroscience research and tips to study better.
	14#	Matute, Helena	The mind trick us: bias and errors we made (A mente engana-nos: desvios e erros cognitivos que todos cometemos)	With the starting point of “we don’t think without errors”, this book analyses the bias and distortions of the human mind. Examples from daily life are analyzed by research in psychology.
	15	Ibánez, Álvaro Ferández	The SharpBrains guide to brain fitness: how to optimize brain health and performance at any age (Como investir no seu cérebro?)	This edition combines a user-friendly tutorial on how the brain works with advice on how to choose and integrate lifestyle changes and research-based brain training. Featuring an analysis of hundreds of scientific studies published in the last 10 years, the book also includes in-depth interviews with 20 leading scientists about brain health thinking and care.
	**16**	Sigman, Mariano	The secret life of the mind: how your brain thinks, feels, and decides (A vida secreta da mente: o nosso cérebro quando decidimos, sentimos e pensamos)	Draws on research in physics, linguistics, psychology, education, and beyond to explain why people who speak more than one language are less prone to dementia; how infants can recognize by sight objects they’ve previously only touched; how babies have an innate sense of right and wrong, even before words; and how we can “read” the thoughts of vegetative patients by decoding patterns in their brain activity.
2019	17#	Caruana, Fausto	The empathic brain (O cérebro empático: como funciona a compreensão do outro?)	Philosophy established the concept of empathy at the beginning of the twentieth century. How the idea evolved to our days? This book review different models that look at empathy and uncover the biological mechanisms that underlie this process.
	18#	Bote, Rubén Moreno	How we make decisions (Como tomamos decisões: os mecanismos neuronais da escolha)	Even the most minor decision uses different neural paths and complex operations in the biology of decision making. This book collects scientific evidence about decision-making and is a contributor to a more profound understanding of some errors we made.
	19#	Domínguez, Daniel Gómez	Math and neuroscience (Matemática e neurociência)	From the ability to count and our numeric system to specific algorithms, we have a mystery: how do we deal with this complexity? This book aims to study the neurologic basis of our number sense and the connection to math.
	**20**#	Garcia, Emílio García	We are our memory (Somos a nossa memória)	Explores the complexity of the memory systems and how they affect our human life.
	21#	Viosca, José	Extraordinary minds (Mentes prodigiosas: fundamentos psicológicos e neuronais das capacidades excecionais)	What happened in the brains of Einstein, Mozart, or Curie? There are persons with an extraordinary capability in a specific filed, how they mind worked? What are the limits of the human mind? This book tries to answer these questions with reflections about scientific research.
	**22**#	Quintero del Álamo, Javier	The teenage brain (O cérebro adolescente: uma mente em construção)	This book explores neuroscientific research about the adolescent brain and transformations during puberty. It aims to inform the reader about behavior typical in this age and neurological changes.
	23#	Lauffer, Javier Correas	Pleasure and reward (Prazer e recompensa: os mecanismos da motivação)	This book is about the role of dopamine in the nervous system and its connection to human behavior. It also analyzes everyday habits like using social networks (like Instagram or Facebook) in the light of neuroscientific research.
	24#	Burgaya-Márquez, Ferran	Does the brain have a sex? Desire, gender, and sexual identity (O cérebro tem sexo? Desejo, género e identidade sexual)	This book is about how our brains work (learning mechanisms, memory) and contribute to human sexuality. This research also connects this data with the concepts of sex and gender.
	**25**#	Sepulcre, Jorge	Neural networks and functional plasticity (Redes cerebrais e plasticidade funcional: o cérebro que se modifica e se adapta)	The structure of the brain, connectivity, and network theory.
	**26**#	Dierssen, Mara	How the brain learns and remembers? [Como aprende (e recorda) o cérebro? Princípios de neurociência para aplicar à educação]	An introduction to neurobiology learning for the general public.
	27#	Maojo, Víctor	Brain and music (Cérebro e música: entre a neurociência, a tecnologia e a arte)	How the brain reacts to music and how it interprets.
	28#	Canessa, Nicola	Reason’s dream: how the brain works [O sonho da razão: como funciona o cérebro]	Introduction to neuroscience for a new audience. New translation with scientific revision.
	**29**#	Cotrufo, Tiziana	In the child’s mind [Na mente da criança: o cérebro nos primeiros anos]	It presents data from research about the development of the human brain in the first years of life. There is a particular highlight to the research about “critical periods” in learning competencies as language and math and the development of memory.
	30#	Tafet, Gustavo E.	Stress: what it is and how it affects us? (Stress: o que é e como nos afeta)	An exploratory text about understanding stress at a psychological and neurological level. New translation with scientific revision.
	**31**#	Alonso, Tomás Ortiz	Neuroscience at home: more than homework (Neurociência em casa: mais do que os trabalhos escolares)	To fill in the gap between neuroscience and education, connect research about learning processes related to school and highlight main evidence.
	**32**#	Berardi, Nicoletta	Environment, plasticity, and brain development (Ambiente, plasticidade e desenvolvimento cerebral)	A book about the role of the environment in neural development.
	33	Daphna, Joe	Gender mosaic (Cérebro e género)	It addresses a controversial topic theme linked to sex differences in the brain explains why there is no such thing as a male or female brain and no neural basis for differentiating people based on sex.
2020	**34**	Castro Caldas, Alexandre; Rato, Joana	Neuromyths (Neuromitos. Ou o que realmente sabemos sobre como funciona o nosso cérebro)	Myths about the mind and the brain are spread across the world. The authors explore each neuromyth and debunk them with evidence gathered in scientific research.
	**35**	Correia, Patrícia; Fonseca, Ana Rita.	The book of the brain: find out what’s inside of your head (O livro do cérebro: descobre o que vai na tua cabeça)	Containing several illustrations and schematic information about the brain anatomy and function was developed for children, but present useful content for all ages.

*#Belongs to a book collection. Bold contributes to bridging neuroscience to education.*

Of the 35 books in review, we selected those that explicitly bridged the gap between neuroscience and education by addressing, in simple language, important themes, such as brain plasticity, types of memory, and the brain of teenagers, demystified some erroneous concepts, or explored the potential of interdisciplinary knowledge of applications in the classroom (marked in bold in Table 3). Of these 35, we highlighted 18 books, written or reviewed by experts, that fulfill the outreach function by disseminating in a useful and clear way, brain content to the educational community. Topics, such as the construction of memories and the relationship between emotions and learning, are the most frequently discussed in these publications and we also found two books that explicitly aim to debunk neuromyths (11 and 34 in Table 3).

## Discussion

Concerns about the academic skills of prospective teacher candidates are recurrently featured in education quality policy discussions around the world (OECD, 2019). Portugal is not an exception, and with this study, we aimed to take a picture of the state of curriculum programs in terms of the presence of up-to-date content about the learning brain. Results showed that teaching training includes scientific domains related to the psychology of education and development, being these the most representative of the mind-brain domains searched. However, we did not directly connect with neuroscientific knowledge since the syllabus analyzed does not evidence this domain, either in the content or in the recommended bibliographical references. Curricular units linked to human biology and psycholinguistics were also identified, but far from representative, given the sparse number of units with the majority being proposed as optional.

Despite the psychology domain content presence, brain knowledge has been deeply absent from the Portuguese teacher’s initial training. With few recent bibliography lists, the historical foundations of psychology and the classic models of child development (e.g., Piaget and Vygotsky theories) are the topics mostly present in the psychology disciplines examined. Also, the revised syllabus does not address the mind, brain, and education integrated view, not even in a slight way. This is in line with the National Council on Teacher Quality report, which indicates that most of the teacher education courses and textbooks in the United States do not cover principles from cognitive psychology related to evidence-based learning, and some of them propagate learning misunderstandings (Pomerance et al., 2016).

Trying to implement popularized strategies, such as the multiple intelligences, the learning styles, or the brain gym, may not be the best use of teacher’s time and, as Weinstein and Sumeracki (2019), we also believed that teachers and researchers can benefit from an open dialogue about the learning science research evidence. It is not new the suggestion that curricular subjects, such as psychology, neuropsychology, and neuroscience in a course syllabus, are one possible way to narrow the knowledge gap concerning how the mind works (Damasio, 2008). According to Liebowitz et al. (2018), the coursework should efficiently introduce key theory embedded into learning sciences, while primarily supporting teaching candidates in building skills in response to the realities they face in their classrooms. However, by our results, Portuguese teachers may be still far from this achievement, especially concerning updating neuroscientific knowledge applied to education.

Providing new tools drawn from scientific research does not have to go through the prescription way (Brookman-Byrne and Thomas, 2018). Involving teachers at the early stages of research projects, shaped by their needs, could help them choose the most appropriate method for a given scenario in their classroom. The Portuguese teacher’s profile recommends that in their professional activity teachers should participate in research projects related to teaching, learning, and student development (DRE, 2001), but analyzing the contents that are worked on in initial training, it seems difficult for these teachers to feel prepared to execute projects on the subject of mind, brain, and education.

Previous studies revealed that Portuguese teachers are very interested in training in these domains and the lack of scientific literacy can contribute to their easily succumbing to neuromyths (Rato et al., 2011, 2013). If on the one hand, we have teachers fascinated to learn more about how the brain works, on the other hand, teacher training itself is losing demand. Our data review of the ITT courses suggests a growing lack of interest to follow a teaching career since these courses are getting low-grade students and have lost candidates over time. Adding to this scenario, we noted that only 2% of Portuguese students express a desire to be a teacher in the PISA report (5% on the OECD average), which are also the ones with low rankings in literacy and mathematics (OECD, 2015). These are enough reasons to make us conclude that the social devaluation of the teaching profession in Portugal is currently a reality.

If the interest in the educational vocation were equal to the general fascination with neuroeducation topics, we would no longer have a problem to solve. Our results show an increase in the number of publications about brain discoveries in the last 6 years. As for the book category, there were more outreach books found, although brain stimulation exercise books were higher produced, compared with textbooks. We also noticed that the available books in European Portuguese are mostly translations, with few Portuguese academics specialized in writing books for the general public. Furthermore, not all of the brain outreach books reviewed are written by experts in the neuroscience field, which also makes this kind of publication more vulnerable to speculation (i.e., the spread of pseudoscience/myths). Nevertheless, the publications reviewed that have school teachers as a target, and which main subjects are addressed to bridge the interdisciplinary area of the mind, brain, and education, remain scarce. As such, it is not run out yet the production of these materials for the educational audience with a scientific quality label. We also realized that even Portuguese researchers with an expressive scientific contribution in the field of relationship between brain sciences and education may not have science extension materials recently published (e.g., Morais, 1997) or authors who may not have the proper recognition in Portugal since their books remain untranslated into Portuguese so far (e.g., Tokuhama-Espinosa, 2018; Dehaene, 2020).

We knew that neuroscience has a major presence in psychology than in education research (Bruer, 2016), and we also knew that the psychology literature has been playing a fundamental role to inform educational settings (Mason, 2009). But, none of this seems enough to reach an interdisciplinary knowledge dialogue, which is structural to the educational success of neuroscience as a field. The brain research contribution, jointly with extensive dissemination and translational work, is increasingly needed to an integrated learning research enterprise for school best practices.

### Limitations and Future Work

As limitations, we highlighted the restraint on public data that when not available unallowed to draw strong conclusions regarding the recommended bibliography in the reviewed courses. However, we also recognized that there may be a wide variability since the same content can be approached differently depending on the training or updating of teachers, which may be a good indicator to explore in future studies. Another weakness is due to the library database used since it is not prepared for the application of typical procedures on a scientific basis (e.g., refined filters and data export), making advanced surveys less accurate and reliant on manual final verification. Also, due to not achieving a full reading and a fact-check, the scientific quality of the reviewed books was based on the broad subjects and authorship expertise, so further studies are required to thoroughly analyze the neuro-prefix materials and workshops or other events that enter schools. A review of the publications to the general public by panel-of-expert would help to distinguish what is pseudoscience from the issues covered in a scientifically supported way.

## Conclusion

The main contribution of the study was to present an exhaustive curricula picture in future training of teachers on Early Childhood Education and Elementary Education and the brain-mind outreach books published in Portugal. The recent explosion of mass-produced information about brain discoveries runs counter to what we see embodied in teaching curricula. Improving the units within the general education category in the ITT courses with an integrated mind-brain-education updated program appears urgent and a possible path to stop misinformation and the spread of educational practices so-called based on the brain but without scientific evidence. Achieving EBPs in schools also involves preparing the educational professionals for scientific literacy right from the beginning of their training. The teacher preparation programs should be seen as a good investment and here neuroscience can play a modest, but booster role in building an evidence-based learning education culture. Still few reference researchers who work on the relationship between brain sciences and education published outreach books for Portuguese educational communities. Quality science communication publications can narrow the scientific brain knowledge gap in educational professionals but, in this case, is dependent on their interest and careful interpretation of this kind of literature.

## Data Availability Statement

The original contributions presented in the study are included in the article/Supplementary Material, further inquiries can be directed to the corresponding authors.

## Ethics Statement

Research protocol was approved by the Comissão de Ética para a Saúde (CES)—Universidade Católica Portuguesa (ref. number 131/2021). Written informed consent was not required in this study in accordance with the national legislation and the institutional requirements.

## Author Contributions

JR and AC-C outlined the research idea. JA collected and processed the data. All authors contributed to the writing and reviewing of the manuscript.

## Conflict of Interest

The authors declare that the research was conducted in the absence of any commercial or financial relationships that could be construed as a potential conflict of interest.

## Publisher’s Note

All claims expressed in this article are solely those of the authors and do not necessarily represent those of their affiliated organizations, or those of the publisher, the editors and the reviewers. Any product that may be evaluated in this article, or claim that may be made by its manufacturer, is not guaranteed or endorsed by the publisher.

## Footnotes

1
www.imbes.org

2
https://www.dges.gov.pt/

3
http://catalogo.bnportugal.gov.pt/
