# Supplementary material for: Looking for the Brain Inside the Initial Teacher Training and Outreach Books in Portugal
Source: Front Psychol. 2022 Feb 28;13:737136. doi: 10.3389/fpsyg.2022.737136 (PMC8919961; doi:10.3389/fpsyg.2022.737136)
Supplement: Supplementary file 1 [file Data_Sheet_1.PDF]

Appendix. List of the ITT courses webpages consulted (school year 2020/21).

		Higher school institution	Webpage
Public	1	University of Algarve	https://esec.ualg.pt/curso/1483
	2	University of the Azores - Faculty of Social and Human Sciences	https://uac.pt/ensino/curso.php?id=4089&l=PT&a=2020/2021&f=FCSH
	3	University of Aveiro	https://www.ua.pt/pt/curso/94
	4	University of Évora – School of Social Sciences	https://www.uevora.pt/estudar/cursos/licenciaturas?curso=2327
	5	University of Madeira – Faculty of Social Sciences	https://www.uma.pt/ensino/1o-ciclo/licenciatura-em-educacao-basica/
	6	University of Minho	https://www.ie.uminho.pt/pt/Ensino/licenciaturas/Paginas/LicenciaturaemEducacaoBasica.aspx
	7	University of Trás-os-Montes e Alto Douro	https://www.utad.pt/estudar/cursos/educacao-basica/
	8	Polytechnic Institute of Beja – Higher School of Education	https://www.ipbeja.pt/cursos/ese-edb/Paginas/default.aspx
	9	Polytechnic Institute of Bragança	http://portal3.ipb.pt/index.php/pt/guiaects/cursos/licenciaturas/curso?cod_escola=3042&cod_curso=9853
	10	Polytechnic Institute of Castelo Branco	https://www.ipcb.pt/esecb/ensino/licenciatura-em-educacao-basica
	11	Polytechnic Institute of Coimbra	https://www.ipc.pt/pt/estudar/cursos/20567
	12	Polytechnic Institute of Guarda	http://www.esecd.ipg.pt/ensino_licenciatura.aspx?id=14&curso=Educa%C3%A7%C3%A3o%20B%C3%A1sica
	13	Polytechnic Institute of Leiria - Higher School of Education	https://www.ipleiria.pt/curso/licenciatura-em-educacao-basica/
	14	Polytechnic Institute of Lisboa – Lisbon Education College	https://www.eselx.ipl.pt/curso/licenciatura/educacao-basica
	15	Polytechnic Institute of Portalegre	https://www.ipportalegre.pt/pt/oferta-formativa/educacao-basica
	16	Polytechnic Institute of Porto - Higher School of Education	https://www.ese.ipp.pt/cursos/licenciatura/461
	17	Polytechnic Institute of Santarém - Higher School of Education	https://siese.ipsantarem.pt/ese/cursos_geral.FormView?P_CUR_SIGLA=LEBA1
	18	Polytechnic Institute of Setúbal	https://www.si.ips.pt/ese_si/cursos_geral.formview?P_CUR_SIGLA=LEB
	19	Polytechnic Institute of Viana do Castelo	https://www.ipvc.pt/cursos/educacao-basica-2/
	20	Polytechnic Institute of Viseu – Higher School of Education	https://www.esev.ipv.pt/Cursos/apresentacao.aspx?value=5
Private			
	1	ISPA – Univ. Institute of Psychological, Social and Life Sciences	https://www.ispa.pt/oferta-formativa/licenciatura-educacao-basica/
	2	Higher School of Education of Fafe	https://www.iesfafe.pt/index.php?option=com_content&view=article&id=9:educacao-basica&catid=5&Itemid=&lang=pt
	3	Jean Piaget Higher School of Education of Arcozelo	https://ipiaget.org/licenciaturas/educacao-basica/
	4	João de Deus Higher school of Education	http://www.joaodedeus.pt/curso/index.asp?id_cnt=20
	5	Paula Frassinetti Higher School of Education	https://www.esepf.pt/licenciatura-em-educacao-basica/
	6	Jean Piaget Higher School of Education of Almada	https://ipiaget.org/licenciaturas/educacao-basica/
	7	Polytechnic Institute of Lusofonia – Higher School of Education	https://www.ipluso.pt/licenciaturas/licenciatura-em-educacao-basica
	8	Higher Institute of Educational Sciences	https://www.isce.pt/pt/estudar/licenciaturas/educacao-basica
	9	Higher Institute of Educational Sciences of Douro	https://www.iscedouro.pt/pt/Oferta-formativa/1-%C2%BA-Ciclo-Licenciaturas/Educacao-Basica/
	10	ISEC Lisboa – Higher Institute of Education and Sciences	https://www.iseclisboa.pt/index.php/pt/cursos/licenciaturas/educacao-basica
